# Symptom management strategies used by older community-dwelling people with multimorbidity and a high symptom burden - a qualitative study

**DOI:** 10.1186/s12877-020-01602-y

**Published:** 2020-06-15

**Authors:** Jeanette Eckerblad, Nana Waldréus, Åsa Johansson Stark, Lisa Ring Jacobsson

**Affiliations:** Department of Neurobiology, Care Sciences and Society, Karolinska Institutet, Alfred Nobels Allé 23, SE-141 83 Huddinge, Stockholm, Sweden

**Keywords:** Content analysis, Multimorbidity, Older people, Symptom management

## Abstract

**Background:**

Older community-dwelling people with multimorbidity are often not only vulnerable, but also suffer from several conditions that could produce a multiplicity of symptoms. This results in a high symptom burden and a reduced health-related quality of life. Even though these individuals often have frequent contact with healthcare providers they are expected to manage both appropriate disease control and symptoms by themselves or with the support of caregivers. The aim of this study was therefore to describe the symptom management strategies used by older community-dwelling people with multimorbidity and a high symptom burden.

**Method:**

A qualitative descriptive design using face-to-face interviews with 20 community-dwelling older people with multimorbidity, a high healthcare consumption and a high symptom burden. People ≥75 years, who had been hospitalized ≥3 times during the previous year, ≥ 3 diagnoses in their medical records and lived at home were included. The participants were between 79 and 89 years old. Data were analysed using content analyses.

**Result:**

Two main strategy categories were found: active symptom management and passive symptom management. The active strategies include the subcategories; *to plan, to distract, to get assistance* and *to use facilitating techniques.* An active strategy meant that participants took matters in their own hands, they could often describe the source of the symptoms and they felt that they had the power to do something to ease their symptoms. A passive symptom management strategy includes the subcategories *to give in* and *to endure*. These subcategories often reflected an inability to describe the source of the symptoms as well as the experience of having no alternative other than passively waiting it out.

**Conclusions:**

These findings show that older people with multimorbidity and a high symptom burden employ various symptom management strategies on daily basis. They had adopted appropriate strategies based on their own experience and knowledge. Healthcare professionals might facilitate daily life for older people with multimorbidity by providing guidance on active management strategies with focus on patient’s own experience and preferences.

## Background

Globally, the population is aging [1, 2] and in some countries, such as Sweden, 25% of the population is older than 60 years of age [3]; moreover, the group of “oldest old”, people older than 80 years, is increasing [2, 4, 5]. Approximately 50–80% of people older than 65 years, and more than 70% of people older than 80 years, have been reported to suffer from multimorbidity, meaning that they have two or more concomitant medical diagnoses [6]. Multimorbidity has so far been associated with high healthcare consumption, including hospitalization and longer hospital stays, institutionalization and healthcare costs, loss of physical functioning, depression, polypharmacy, lower health-related quality of life (HrQoL), a high symptom burden and high mortality [7–10]. Chronic disease among older people is considered to be one of the largest healthcare challenges of this century, affecting both socioeconomics and the healthcare systems [11–13]. To date, healthcare organizations have not been adequately prepared or designed to meet the challenges of older people with multimorbidity [14, 15].

One of the public health goals for this aging society is to reduce the impact of chronic diseases [11–13]. Notwithstanding, there is still a paucity of research addressing the prevalence and management strategies of concurrent symptoms and symptom burden in older people with multimorbidity, even though this is a frequent and well-known clinical problem [16, 17]. People with multimorbidity report a large variation in symptoms, of which pain, dry mouth, lack of energy, and numbness/tingling in hands/feet were reported by 50% or more [10].

According to the Symptom Management Theory, the goal of symptom management is to prevent or delay the negative outcomes of symptoms through biomedical, professional and management strategies (Dodd, 2001). In community-dwelling people diagnosed with and treated for chronic diseases, the responsibility of managing symptoms on a day-to-day basis often rests with the individuals themselves [18, 19]. However, all troublesome symptoms require proper assessment and management in order to prevent, delay, or minimize a high symptom burden [19].

With a growing number of older people living at home, more knowledge on symptom management is needed to facilitate better delivery of appropriate healthcare [20]. To gain further insight into the phenomenon of living with a high symptom burden, the aim of this study was to describe the symptom management strategies used by older community-dwelling people with multimorbidity and a high symptom burden.

## Methods

### Design

A qualitative descriptive design using face-to-face interviews with 20 community-dwelling older people with multimorbidity and a high symptom burden. The participants were engaged in a prospective, single centre, randomized, controlled trial, the Ambulatory Geriatric Assessment: A Frailty Intervention Trail (AGe-FIT) [21].

The AGe-FIT included a selected group of 382 older people with multimorbidity and a high healthcare consumption. People ≥75 years, who had been hospitalized ≥3 times during the previous year, who had ≥3 diagnoses in their medical records according to the International classification of diseases (ICD-10) and who lived at home were included. Living in a nursing home were excluded [21, 22]. The participants were recruited through the patient administrative system of the County Council.

Results from AGe-FIT had shown that older people with multimorbidity had reported a high symptom burden on the Memorial Symptom Assessment Scale (MSAS) [10] this triggered an interest to further describe the experiences of living with a high symptom burden [23] and to explore what symptom management strategies older people with multimorbidity use. Data from the qualitative interviews have previously been analysed and published in an article focusing on the experience of living with a high symptom burden [23]. The present study focuses on symptom management strategies. All parts of the study followed the ethical guidelines given in the Declaration of Helsinki and the study was approved by the Linkoping Regional Ethical Review Board (Dnr 2012/244–32).

### Study settings

This study was conducted in Sweden where the county council and municipality are responsible for the provision of health and social care, funded by income taxes. The municipality provides home health and social care, including care provision nursing homes when needed. Home care typically includes home help services to support older people in conducting activities of daily living (ADL) and instrumental ADL. Participants were recruited from a middle-sized city (120000) in the south-east of Sweden where approximately 9% of the inhabitants are 75 years or older. In this community, health care is mainly provided by the primary care centers and one general hospital.

### Participants and procedure

All participants in this study were recruited consecutively during the second-year follow-up of the Age-FIT [21]. In this study, we sought a purposive sample, only including participants having a high symptom burden, i.e. a high score on the MSAS.

The MSAS was used to describe the participants’ symptom burden [24]. The MSAS includes 32 different symptoms, a four-point rating scale for severity and frequency (1–4), and five-points for distress (min-max 0–4). Participants who had reported a high score on the MSAS (scores ≥3 per symptom on frequency, severity or distress in at least four prevalent symptoms) during the second-year follow-up of the AGe-FIT were included. No significant differences between the intervention and control group regarding symptom burden were found [25]. Participants from both groups were included in this study.

Study participants who fulfilled the inclusion criteria were sent an information letter explaining the purpose of the study immediately after completing the second-year follow-up. Twenty patients with mean age of 84, (SD ±2.9) were included, 16 women and 4 men (Table 1). Fifteen of the 20 participants lived alone and of those 14 were widows and one divorced. Five were married. All participants lived in their own apartments and had contact with health care. All needed home care and/or home help services to varying degrees from the municipality or next of kin.

**Table 1:** Background characteristics

	***n*** = 20
Age (yrs), mean (SD)	84 (±2.9)
Women n (%)	16 (80)
Lived alone n (%)	15 (75)
Poor hearing with or without hearing device n (%)	7 (35)
Poor vision with or without glasses n (%)	3 (15)
Diagnosis according to ICD-10 Chapter	
01. Certain infectious and parasitic diseases (A00-B99) n (%)	5 (25)
02. Neoplasms (C00-D48) n (%)	9 (45)
03. Diseases of the blood and blood-forming organs and certain disorders involving the immune mechanism (D50-D89) n (%)	5 (25)
04. Endocrine, nutritional and metabolic diseases (E00-E90) n (%)	12 (60)
05. Mental and behavioural disorders (F00-F99) n (%)	10 (50)
06. Diseases of the nervous system (G00-G99) n (%)	10 (50)
07. Diseases of the eye and adnexa (H00-H59) n (%)	14 (70)
08. Diseases of the ear and mastoid process (H60-H95) n (%)	7 (35)
09. Diseases of the circulatory system (I00-I99) n (%)	20 (100)
10. Diseases of the respiratory system (J00-J99) n (%)	11 (55)
11. Diseases of the digestive system (K00-K93) n (%)	10 (50)
12. Diseases of the skin and subcutaneous tissue (L00-L99) n (%)	9 (45)
13. Diseases of the musculoskeletal system and connective tissue (M00-M99) n (%)	19 (95)

*ICD* International Classification of Diseases

The participants had a high total symptom burden score, median 0.96 (range 0.31–2.27) (min-max 0–4) and a mean of 12 (±5.3) prevalent symptoms per person [10]. Each participant experienced a large variation in symptoms (Table 2).

**Table 2:** MSAS symptom burden score of older people who reported the symptom as present during the preceding week

Symptom	Number of participants who reported the symptom *n* = 20	Symptom burden score range 0.90–4.0	(±SD)
Lack of energy	18	2.8	(±0.75)
Pain	17	2.9	(±0.62)
Dry mouth	16	2.7	(±0.92)
Feeling drowsy	14	2.1	(±0.67)
Difficulty sleeping	13	3.0	(±0.80)
Worrying	11	2.7	(±0.92)
Swelling of arms or legs	11	2.4	(±1.19)
Numbness/tingling in hands/feet	10	2.8	(±0.55)
Feeling bloated	10	3.0	(±0.47)
Shortness of breath	10	3.7	(±0.55)
Dizziness	10	2.2	(±0.81)
Problems with urination	9	2.8	(±0.82)
Feeling sad	8	3.0	(±0.65)
Cough	8	2.2	(±0.74)
Lack of appetite	7	2.3	(±0.64)
Feeling irritable	7	2.1	(±0.79)
Itching	6	2.4	(±1.10)
Feeling nervous	6	2.9	(±0.98)
Difficulty concentrating	6	2.6	(±0.61)
Diarrhoea	5	2.8	(±0.58)
“I don’t look like myself”	5	2.7	(±0.97)
Sweats	4	1.8	(±0.57)
Difficulty swallowing	4	3.0	(±0.98)
Mouth sores	4	2.5	(±0.71)
Changes in skin	4	1.3	(±1.14)
Constipation	4	2.2	(±0.91)
Problems with sexual interest/activity	3	2.0	(±0.91)
Weight loss	3	1.6	(±1.21)
Nausea	3	1.8	(±0.38)
Change in the way food tastes	2	3.0	(±1.41)
Hair loss	2	3.8	(±0.35)
Vomiting	0		

The MSAS symptom burden score is the mean score of the three dimensions: frequency, severity and distress (range 0–4.0)

### Data collection

All interviews were performed by the first author (JE), RN, PhD with previous experience and formal education of performing qualitative interviews. The first interview was performed as a pilot, no changes in the interview guide were made (Additional file 1). All interviews were performed between March and September 2013. The MSAS score from each participant’s most recent data collection was used to guide the interviews and each symptom was discussed one-by-one with the participants. During the interviews, the participants were asked to describe -the status of their symptoms since the last data collection, −what they did to manage that symptom, −how they had learned that management strategy and -how well that strategy worked. All interviews took place at the participants’ homes and the visit lasted approximately 2 hours with small talk; the interviews ranged between 20 and 55 min. All interviews were audio-recorded and transcribed, confidentiality was ensured by allocating a code number to each interview transcript. When 20 interviews were performed, saturation was achieved [26, 27].

### Data analyses

**Fig. 1:**
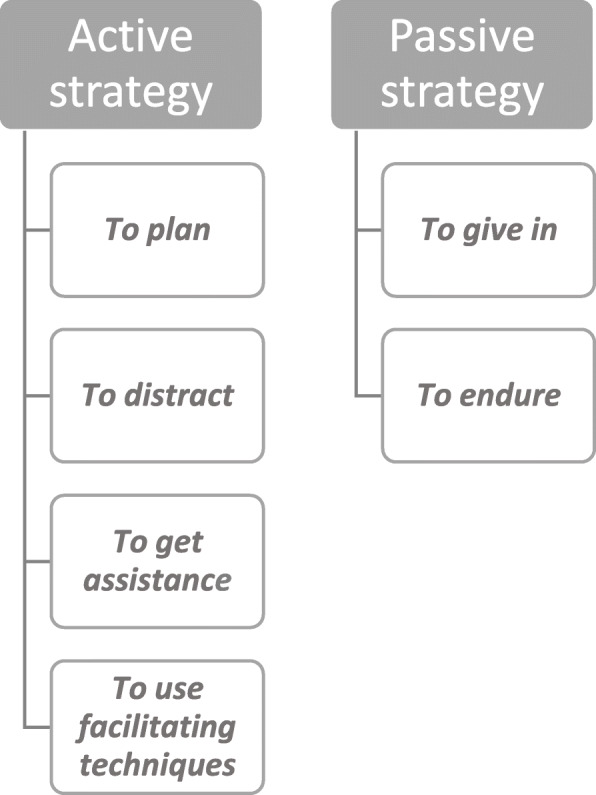
Categories and subcategories of the findings

The analysis was inductive and performed using qualitative content analysis [28]. The transcribed interviews were read through several times, and significant text units (meaning units) were identified, marked and labelled with open coding close to the participant’s statements, and sorted into subcategories depending on the content (Table 3). This primary analysis was performed by two of the authors. The final structure (Fig. 1) was created after all authors had checked and reviewed the data.

**Table 3:** Example of the analytical process

Meaning unit	Condensed meaning unit	Cods	Subcategories	Categories
I am often away from home involved in any of my activities, it’s much better to be around people you know	Commitment and company ease the pain	keeping busy	*To distract*	*Active strategy*
But hopefully it can get better now that I got a shot (cortisone) in the knee	Turned to healthcare in hope of solace	Getting support	*To get assistance*	*Active strategy*

## Result

In accordance with the purposive sample, all participants had many concomitant diagnoses and a high symptom burden. The main strategies used by the participants were active and passive symptom management. The active strategies included, *to plan, to distract, to get assistance* and *to use facilitating techniques,* while the passive strategies included *to give in* and *to endure.*

### Active strategies

When using an active strategy, the participants took matters in their own hands and felt they could do something to ease the symptom. The participants that used an active symptom management strategy could often explain or describe the source of the symptom and why they thought their strategy worked e.g. the circulation of the body was insufficient (strategy: use different physical activities), the body did not get enough oxygen (use different breathing techniques), and the nerves had somehow got trapped in the joints causing pain or tingling in hands or feet (use massage). Although their management strategy did not always make the symptom disappear altogether, it could often ease the burden temporarily or prevent the symptom from getting worse. All participants were receiving pharmacological treatment for many different diseases but, despite this, medication was seldom mentioned as a method for managing their symptoms or relieving symptoms. When asked where they had learned to use those techniques, they often referred to friends, family members, something they had read in a magazine or by trial and error.

#### To plan

The symptoms experienced by the participants were often considered to limit life in different ways and at different levels. Careful planning of activities was an active strategy, often in detail, and was considered crucial if they were to manage symptoms and feel safe: careful pre-planning of the trip, resting before leaving the house, how to get there - would it be possible to walk, go by bus or take a taxi? How long would it take, would there be anywhere to sit down while waiting for transport? Even with careful planning their health could deteriorate rapidly, and everything would have to be cancelled due to an increased symptom burden. Cancelling at the last minute was described as problematic since they felt they let people down and disappointed them. Participants who frequently had to use the toilet could, for example, pre-investigate where to find the nearest toilet or put on an adult diaper - just in case. Another strategy, with the same goal, was to plan when different medications should be taken:

“*Yes, if you're going away, or like yesterday, when we attended a funeral, then I did not dare take diuretics*” (Participant 9; [P]).

People with dizziness could plan where they would best position themselves in a room in the event they lost their balance, fell over, or were forced to crawl to the nearest phone to get help. Before sitting down, they checked that the table and chair appeared steady or robust enough to support them when they got up again. Due to a lack of energy, it was important to limit the activities during a day, and to not be away from home for a long period of time just in case the symptom burden increased. Not traveling great distances was also mentioned as a strategy in the event they had to quickly return home due to an increased symptom burden.

“*But I do not want to travel anywhere far away, but I've been on some shorter trips and it has gone well*” (P 3).

#### To distract

Distraction was frequently used as an active strategy especially for managing pain. Pain was extremely stressful and takes incredible amounts of energy. Therefore, it was considered important to reason with yourself and try to do something that distracts from it and makes time go faster. Distractions included crosswords, lying down and closing your eyes while reciting poems to yourself. Doing some kind of hobby was considered by some to distract from symptoms. Getting out of the home and interacting with others, instead of just being at home monitoring symptoms, was by many participants considered to be the best distraction strategy for managing symptoms:

*“Well if you talk to people you might forget about the pain instead of going home all alone and just feeling it. That's really what makes the difference” (P1).*

Reasoning “*with yourself”* was another distraction. By comparing themselves with others of the same age and situation who had an even greater symptom burden. Own symptoms were experienced easier to bear and manage when they realized that there were those who had significantly more severe symptoms:

“*Well I suppose the body can’t function perfectly at my age, and I see others who look very frail, so in that way I feel I'm privileged*” (P3).

#### To get assistance

Another active strategy was to ask someone for assistance to manage their symptoms. Most often the assistance came from family or the healthcare services, but it could also be help or advice from healthcare professionals. All participants in this study had several chronic diseases necessitating frequent encounters with different healthcare professionals. The travel arrangements just to get to the clinic and back again could be too much of an effort when the symptom burden was high, and they needed assistance to be able to get there at all.

*“The clinic wanted me to come down there, but I had to decline, last time I had to wait over an hour for my ride (car) to get back home. I’m just too exhausted I don’t have the energy to do it” (P 5).*

Some participants had symptoms necessitating assistance from relatives and family on a regular basis. Sometimes they felt forced to ask for help from friends, family or neighbours due to problems associated with the high symptom burden:

*“I have my daughter who assists me with things I no longer manage myself, but it is not easy for her either, she also has her job to think about” (P1).*

#### To use facilitating techniques

One active strategy used by participants to ease the pain or tingling in hands or feet was different types of physical activities. When pain struck, they described it as impossible to lay down or keep still and that moving around to get the circulation going was the best option. The physical activities could involve walking, spinning/cycling or doing squats. Other techniques were to sit up and do breathing exercises in order to get more oxygen into the system.

*“When I get this pain in my chest and back, I cannot go and lie down, that will only make it worse. I’ll often try to do breathing exercises” (P2).*

Participants also used massage, warm socks at night, bandages, breathing exercises, TENS, relaxation, and to lie down in a specific position to ease the pain.

*“Well I figured that, if I rub really hard at this area (over the ankle) it gets warm, and then somehow things seems to loosen up a bit, and the pain goes away” (P3).*

To relieve symptoms of a dry mouth or from the gastrointestinal tract, they tried gargling water, excluded various foods, and tested different non-prescription drugs from the pharmacy.

Most of these exercises and methods they had learned from friends and family or through trial and error.

### Passive strategies

The participants sometimes felt they had no choice when managing symptoms other than to passively give in or to endure. The participants employing this symptom management strategy could not describe the source of their symptom, they often referred to it as “*no one knows, it’s “just the way it is*” or “*the body is just totally worn out*”.

#### To give in

When the participants talked about living with multiple (concomitant) symptoms or being fatigued, they had a passive strategy. They described it as though there is nothing anyone could do about it anyway, and they associated it with old age and the aging body.

*“Well I’m 86 years old so I just have to accept my fate (P 17).*

A very common passive way to manage their symptoms was to sit or lie down to rest. Participants described that they took a rest in order to suppress pain, for example, and that they often experienced situations of total powerlessness.

*“I suffer from a lot of pain, sometimes I start to do something (talks about baking bread), but the pain gets too intense, and I just can’t finish. And when I try to do something else, it is the same story all over again” (P 17).*

The participants sometimes experienced limitations in life that they could not control. In these cases, the only thing left to do was to stay at home instead of going on trips or to gatherings. Things they would like to do were no longer possible and they had to let go of interests in life.

*“I was very active and dedicated once upon a time, but that has all changed now, now I'm more indifferent” (P18).*

#### To endure

For some participants, the symptoms were so severe and debilitating that they had no alternative but to passively try to endure. They described it as a vegetative life, and they tried to cope from 1 day to the next.

*“I am not really alert during the day, I’m mostly a sleep and I cannot really take any initiative of my own // I think I have outlived my own expectation by now” (P7).*

Some participants blamed themselves for not being able to live a normal life. They were annoyed at themselves and considered themselves worthless and a burden for their family.

*“My wife has to do everything around here now, I just sit and do nothing// I cannot even go for a walk outside anymore, my body is worn out” (P19).*

## Discussion

These findings contribute to our understanding of symptom management strategies used by older community-dwelling people with multimorbidity. The participants use a variety of management strategies to ease their symptom burden, which could be either active or passive. The active strategy meant that when the participant experienced an increase in a specific symptom, they used various techniques to manage this. The passive strategy meant that the participant was unable to act or avoided the situation, instead of trying to take charge.

Active and passive symptom management strategies could correspond with problem- and emotion-focused coping earlier described by Lazarus & Folkman [29]. Situations when people think something constructive can be done encourages problem-focused coping, not unlike the active symptom management strategy, while the passive strategy is similar to emotion-focused coping [30]. No studies with older community-dwelling people with multimorbidity have been found reporting active and passive symptom management strategies, nor problem- and emotion-focused coping. However, there are studies within other contexts. Active strategies to manage symptoms has been found to be more common than passive strategies, such as help seeking, altering routines and distraction [31]. Hence, the passive strategies need to be identified, as they might have a negative impact on patients’ health and wellbeing.

The participants in this study managed their symptoms in the best possible way they knew. By using an active strategy, the older person felt empowered and in charge, in contrast to persons using a passive strategy. People with multimorbidity have previously expressed that they shift between experiencing disruption because of the condition, and feelings of being able to accommodate the challenges the condition entails. They described constantly reassessing their prioritization of their condition [32]. We know from an earlier study in this population that people who live with a high symptom burden may have a feeling of being dependent, dejected, inadequate and limited [23].

Some participants felt that the symptoms were so severe and debilitating that they could not manage them at all, they had no alternative but to try and endure. In people with multimorbidity, the combination of physical and emotional symptoms can increase the total symptom burden, which in turn can result in a greater negative impact on daily lives [33]. Healthcare professionals must be part of the discussion if they are to raise questions on symptom management or discuss symptom maintenance to provide support and give advice. This might be important, especially in cases where the participants described that there was nothing they could do because of their old age. A recent study, reporting on adjustment to loss in old age, showed that passive adaption such as avoidance resulted in insufficient accommodation, while physical losses tackled via problem-solving and identification of new alternatives were found to be effective [34]. Passive symptom management strategies, for example to give in, appear to be inadequate whereas a combination of active management strategies, such as solving problems by planning activities and ask for assistance, appears to result in more effective symptom management. Thus, healthcare professionals should explore and support active symptom strategies among older people with multimorbidity.

One common active strategy for symptom management was to get assistance, often provided by family members or the healthcare services. In an individualized society, older people might choose to passively endure, instead of risking the potential of being a burden to their families, while others might prefer the support from society as an alternative [35, 36].

Older people with multimorbidity often have a heavy medication load and are often worried that the medication could be harmful or give unwanted side effects [37]. Therefore, it must be considered important to identify patients at risk of non-adherence to treatment, in order to reduce suffering and develop strategies to enhance adherence [38]. On the other hand, studies have reported that older people might be underdiagnosed and undertreated when it comes to e.g. pain [39, 40].

The importance of supporting self-management for people with multimorbidity has been increasingly highlighted as a key component of improving the overall health of this population [14]. If healthcare professionals are to support and facilitate symptom management in older people with multimorbidity, symptom self-management must be highlighted and recognized. The Symptom Management Theory [18] identifies three dimensions that should be considered for effective symptom management, (symptom experience, symptom management strategies, and symptom outcomes). Shared decision-making has been suggested to be the best way for effective management strategies to be shared between older people with multimorbidity and healthcare professionals [14]. A comprehensive approach, with a focus on personal preferences, careful interpretation of the available evidence, taking time to discuss goals and preferences including the burden of any treatment, is important [17].

The participants in this study described that the symptom could vary from day-to-day, both in occurrence and burden. Symptom management strategies may also need to change as symptoms vary over time. The knowledge of the individual’s own self-management strategy might help healthcare professionals to further encourage and support these symptom management strategies to reduce symptom frequency, minimize symptom severity, and relieve symptom distress. It seems urgent to find those people whose symptoms are so severe and debilitating that they cannot cope with them at all. In these cases, home visits could provide guidance on management strategies and other necessary help.

In order to provide appropriate advice regarding symptom management, it is important that healthcare professionals take the time to listen to the individual’s perception of current abilities at all meetings with the healthcare services. Previous studies have shown that a higher symptom burden is a factor related to a lower HrQoL in older people with multimorbidity resulting in functional impairment, disability, and depression [9, 41]. Accessible psychiatric care, as well as regular follow-up of these patients’ emotional state, have shown to be helpful [33]. Moreover, older people with multimorbidity often have a heavy treatment burden as a result of the several management plans and lifestyle changes prescribed for the various conditions [14].

### Methodological consideration

The design of this study enabled us to gain a deeper understanding of a less-explored subject — symptom management strategies in older people with multimorbidity and high healthcare consumption. This lack of knowledge and evidence relates to the regular exclusion of older people from clinical trials due to their frailty and their several interfering chronic diseases [42]. Older people with multimorbidity are considered a vulnerable group and require special consideration when the subject of research. All interviews were performed in the participant’s own home so that the participant could easily stop their participation at any time; this however did not occur in any of the interviews. To ensure trustworthiness of this study, the concepts of credibility, conformability, dependability and transferability have to be taken into consideration [28]. All authors participated in the final analysis process; this triangulation increases credibility and increased the rigor of the data. The findings are presented with illuminating verbatim quotations to reach confirmability [28].

Some limitations of the study must be considered. Twenty participants were interviewed in this study and even though saturation seemed to be fulfilled, additional symptom management strategies might have emerged if more interviews had been performed. However, the sample size in qualitative studies depends on required information and is often low [27, 43]. The participants interviewed in this study were frail and weak, and some found it difficult to describe their symptom management strategy. Some interviews may have been shorter because of this, and it may have decreased the quality and richness of these interviews. Efforts were made to create optimal conditions for the interviews.

## Conclusions

These findings show that older people with multimorbidity and a high symptom burden monitor their symptoms and use a variety of symptom management strategies on daily basis. They had adopted appropriate symptom management methods based on their own experience and knowledge. Active strategies meant that participants took matters in their own hands, and felt they had the power to ease their symptoms. Passive symptom management strategies however often meant an inability to act, and the experience of having no alternative but to passively give in or to endure. Healthcare professional might facilitate daily life for older people with multimorbidity by providing guidance on management strategies with focus on patient’s own experience and preferences.

## Supplementary information

**Additional file 1.** Interview guide.

## Data Availability

The datasets used and/or analysed during the current study are available from the corresponding author on reasonable request.

## Abbreviations

HrQoL: Health related Quality of Life
MSAS: Memorial Symptom Assessment Scale
P: Participant
