# Supplementary material for: Symptom management strategies used by older community-dwelling people with multimorbidity and a high symptom burden - a qualitative study
Source: BMC Geriatr. 2020 Jun 15;20:210. doi: 10.1186/s12877-020-01602-y (PMC7296961; doi:10.1186/s12877-020-01602-y)
Supplement: Supplementary file 1 — Additional file 1. Interview guide. [file 12877_2020_1602_MOESM1_ESM.docx]

**Interview Guide**

The Memorial symptom assessment scale score from each participant’s most recent data collection was used to guide the interviews, and each symptom was discussed one-by-one with the participants.

-Last week you met my colleague and you filled in a questioner about symptom experience. Now I am here to talk to you about how you manage these symptoms in your everyday life.

-What Symptoms is bothering you the most right now?

-How does that feel?

-How does these symptom effect you?

-What do you do to manage these symptoms?

-Why do you think that works?

-Where did you learn to do that?

**The follow-up questions**

Could you please tell me more?

Could you please describe further?

What did you think in that situation?

How did you handle that?

What did you do then?
